# Supplementary material for: A chiral selectivity relaxed paralog of DTD for proofreading tRNA mischarging in Animalia
Source: Nat Commun. 2018 Feb 6;9:511. doi: 10.1038/s41467-017-02204-w (PMC5802732; doi:10.1038/s41467-017-02204-w)
Supplement: Supplementary file 1 — Supplementary Information [file 41467_2017_2204_MOESM1_ESM.docx]

**Supplementary information**

**
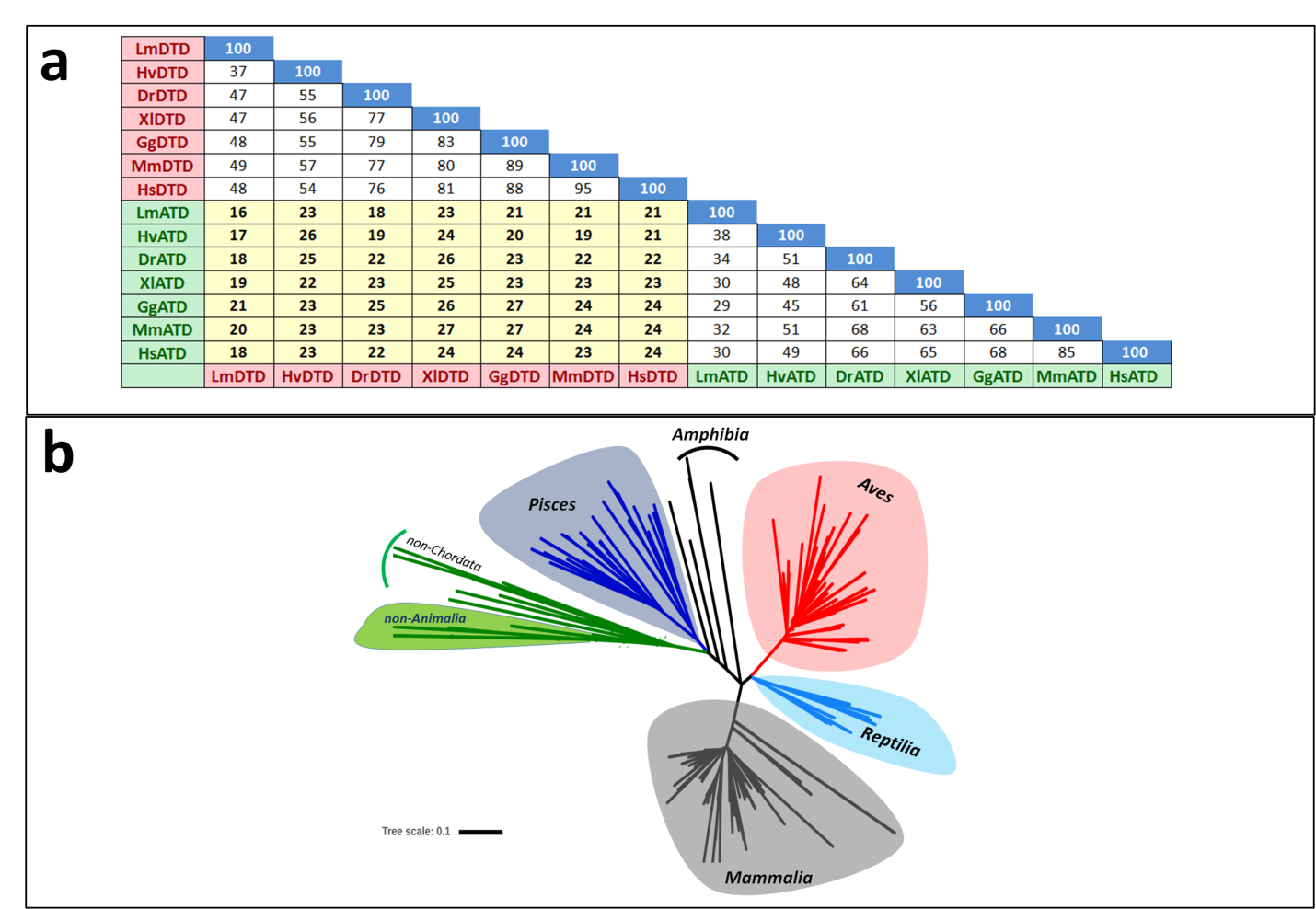
**

**Supplementary Fig. 1** ATD is distinct from DTD present in kingdom Animalia. **a** Matrix showing percentage identities between DTDs, between ATDs as well as between DTDs and ATDs belonging to representative organisms. Lm, *Leishmania major*; Hv, *Hydra vulgaris*; Dr, *Danio rerio*; Xl, *Xenopus laevis*; Gg, *Gallus gallus*; Mm, *Mus musculus*; Hs, *Homo sapiens*. **b** Phylogenetic analysis depicting the presence of ATD mainly in kingdom Animalia, and more specifically in phylum Chordata, which comprises Pisces, Amphibia, Reptilia, Aves and Mammalia. The few non-Animalia that harbor ATD are mostly parasites of various chordates, hence the possibility of horizontal transfer of ATD gene.

**
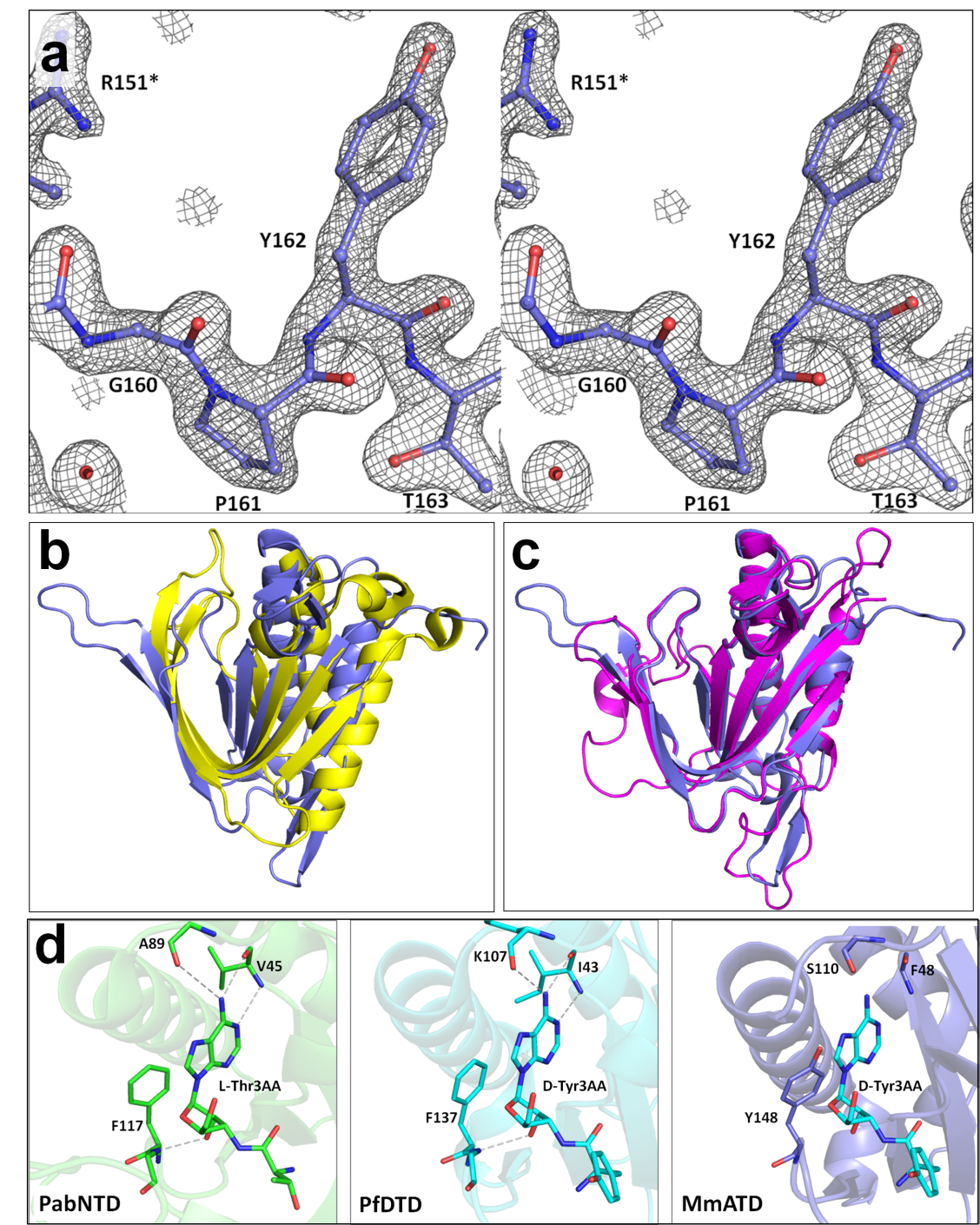
**

**Supplementary Fig. 2** ATD, DTD and NTD are structural homologs belonging to the DTD-like fold. **a** Stereo image of a part of the active site of MmATD showing the (2*F_o_-F_c_*) electron density map contoured at 2.0σ. **b** Structural overlap of MmATD monomer (blue) on PabNTD monomer (yellow; PDB id: 3PD3) (r.m.s.d., 3.34 Å over 77 Cα atoms) showing that the two belong to the same fold. **c** Structural overlap of MmATD monomer (blue) on LmATD monomer (magenta; PDB id: 1TC5) (r.m.s.d., 1.29 Å over 148 Cα atoms). **d** The elements of adenine-binding pocket of the DTD-like fold, encompassing NTD, DTD and ATD, are conserved. The ligand D-tyrosyl-3′-aminoadenosine (D-Tyr3AA) in MmATD has been modeled on the basis of structural overlap of MmATD on PfDTD (PDB id: 4NBI). The PDB id for PabNTD is 3PD3. Residues from the dimeric counterpart are indicated by *.

**
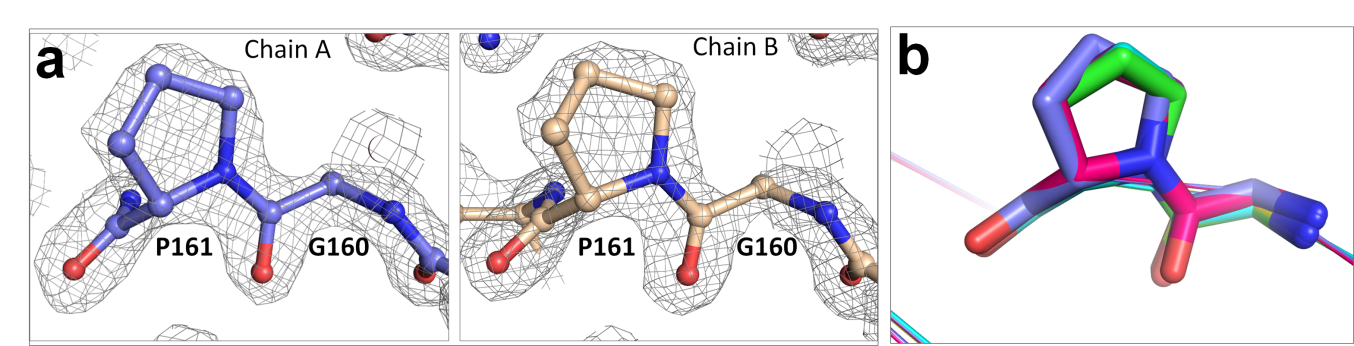
**

**Supplementary Fig. 3** ATD has an active site Gly-*trans*Pro motif. **a** (2*F_o_-F_c_*) map, contoured at 2σ, showing clean density for the Gly-*trans*Pro motif of both protomers present in the asymmetric unit of MmATD crystal structure. **b** Structural superposition of LmATD protomers (cyan, green, purple and yellow; PDB id: 1TC5) on MmATD protomers (blue and magenta) highlighting the *trans* conformation of the active site Gly-Pro motif in both proteins.

**
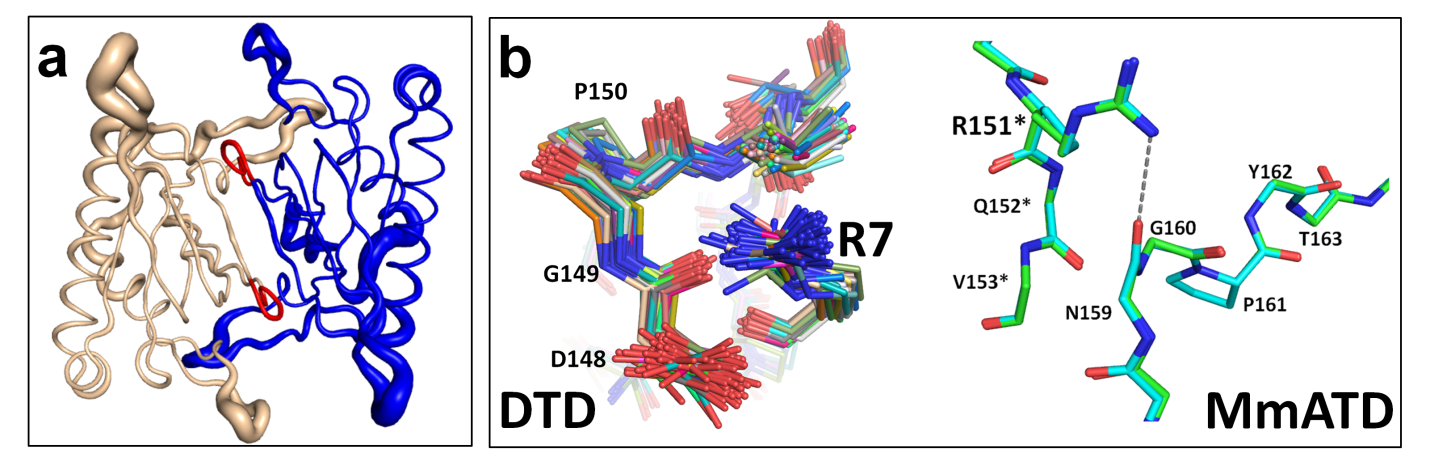
**

**Supplementary Fig. 4** The Gly-*trans*Pro motif in ATD is rigidly held like the Gly-*cis*Pro motif in DTD but with subtle variations. **a** Backbone representation of MmATD showing the variation in atomic B-factor. Regions represented as thin lines are more rigid and therefore have low B-factor values, whereas those depicted as thick lines are more flexible and thus have higher values. The atomic B-factor for the structure lies in the range 18–62 Å^2^. Regions depicted in red represent glycine and proline residues of the Gly-*trans*Pro motif, whose average B-factor is 24 Å^2^ (B-factor of protein is 31 Å^2^). This value is similar to that of PfDTD (B-factors of Gly-*cis*Pro motif and protein are 25 Å^2^ and 29 Å^2^, respectively; PDB id: 4NBI), showing that the Gly-*trans*Pro motif in ATD is rigidly fixed like the Gly-*cis*Pro motif in DTD. The two monomers of ATD homodimer have been rendered in different colours. **b** Structural superposition of 107 protomers of DTD from 19 PDBs and 5 different organisms showing rigid fixation of Gly-*cis*Pro motif by a conserved interaction of Asp148 carbonyl oxygen with the side chain of a highly conserved arginine (Arg7, PfDTD) from the same monomer. Numbering of residues is according to PfDTD. Structural overlap of the two protomers of MmATD showing that Gly-*trans*Pro motif in ATD is firmly fixed by a conserved interaction with the side chain of an invariant arginine (Arg151). Residues from the dimeric counterpart are indicated by *.


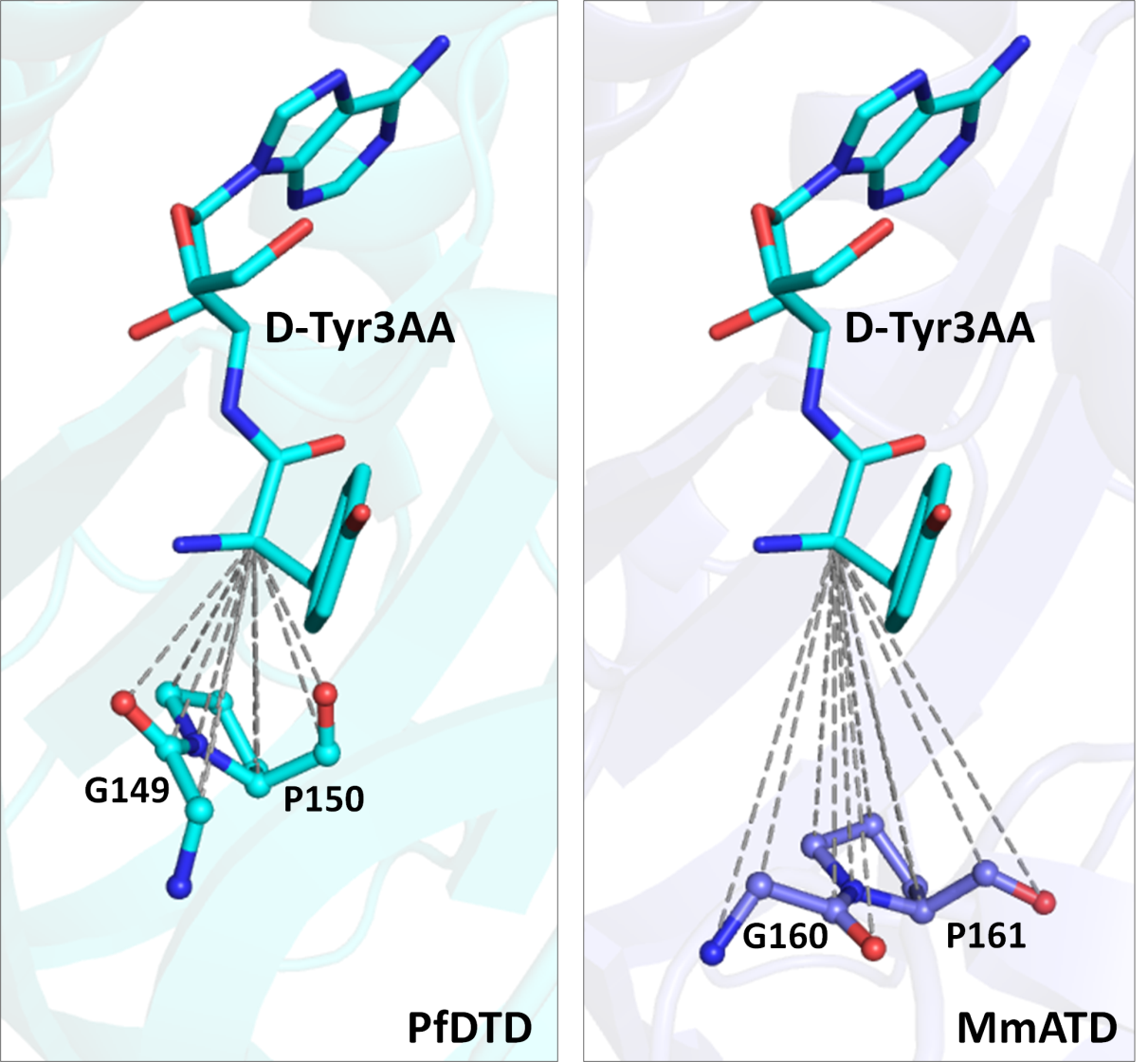


**Supplementary Fig. 5** ATD has “additional” space in its active site pocket compared to DTD. Comparison between active site pockets of PfDTD (PDB id: 4NBI) and MmATD showing “additional” space in the latter due to the inward movement of Gly-Pro carbonyl oxygens. For MmATD, the ligand was modeled in the active site after superposition of MmATD dimer on PfDTD dimer.

**
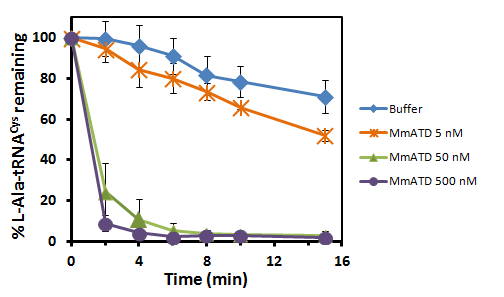
**

**Supplementary Fig. 6** ATD has lower activity on L-Ala-tRNA^Cys^(G4•U69). Deacylation of L-Ala-tRNA^Cys^(G4•U69) by different concentrations of MmATD. The substrate was used at 200 nM final concentration. Error bars denote one standard deviation from the mean of three independent readings.

**
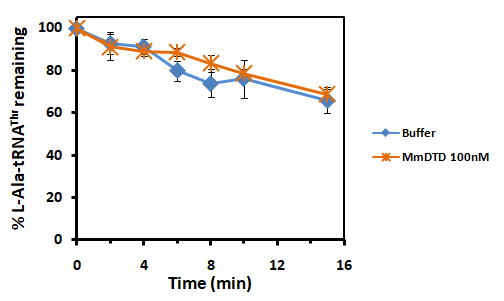
**

**Supplementary Fig. 7** DTD does not act on L-Ala-tRNA^Thr^(G4•U69). Deacylation of L-Ala-tRNA^Thr^(G4•U69) by MmDTD. The substrate was used at 200 nM final concentration. Error bars denote one standard deviation from the mean of three independent readings.

**
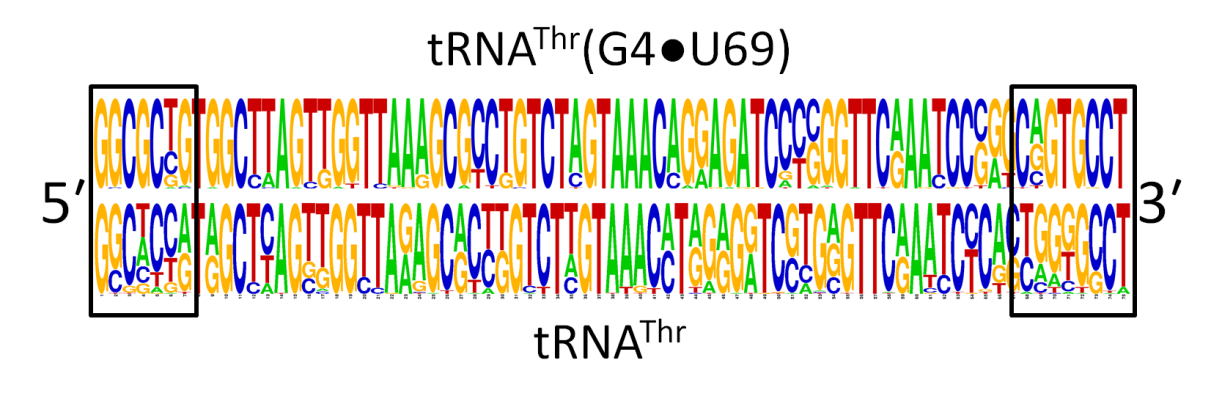
**

**Supplementary Fig. 8** Acceptor stem elements of tRNA^Thr^(G4•U69) genes are highly conserved. Consensus sequence showing significantly higher conservation of acceptor stem residues (enclosed in boxes) in tRNA^Thr^(G4•U69) genes than in non-G4•U69-containing tRNA^Thr^ genes. The gene sequences taken for analysis belong to the following representative organisms: *Strongylocentrotus purpuratus*, *Latimeria chalumnae*, *G. gallus*, *M. musculus*, *H. sapiens*.

**Supplementary Table 1 “Additional” space in ATD’s active site pocket.** Comparison of distances between atoms of Gly-Pro residues and Cα of the ligand D-Tyr3AA for PfDTD (PDB id: 4NBI) and MmATD^†^.

| **Gly-*cis*Pro**  **(PfDTD)** | **Distance in Å**  **(DTD)** | | **Atom** | **Distance in Å**  **(ATD)** | | **Gly-*trans*Pro**  **(MmATD**^†^**)** |
| --- | --- | --- | --- | --- | --- | --- |
|  | **Monomer** | |  | **Monomer** | |  |
|  | **A** | **B** |  | **A** | **B** |  |
| **Gly149** | **6.5** | **6.3** | **N** | **7.7** | **7.8** | **Gly160** |
|  | **5.1** | **4.9** | **Cα** | **6.4** | **6.5** |  |
|  | **4.2** | **4.1** | **C′** | **6.7** | **6.9** |  |
|  | **3.8** | **3.7** | **O** | **7.3** | **7.4** |  |
| **Pro150** | **4.3** | **4.3** | **N** | **6.8** | **7.0** | **Pro161** |
|  | **5.1** | **5.1** | **Cα** | **7.7** | **7.8** |  |
|  | **4.6** | **4.5** | **C′** | **7.2** | **7.2** |  |
|  | **3.4** | **3.3** | **O** | **8.1** | **8.2** |  |
|  | **5.7** | **5.8** | **Cβ** | **8.3** | **8.3** |  |
|  | **4.9** | **5.2** | **Cγ** | **7.5** | **7.2** |  |
|  | **4.3** | **4.4** | **Cδ** | **6.8** | **6.8** |  |

† For MmATD, the distances were calculated by modelling the ligand in the active site after superposition of MmATD dimer on PfDTD dimer.

**Supplementary Table 2 Substrate specificity of ATD.** Comparison of MmATD-mediated apparent deacylation rates (*k*_obs_) of various aminoacyl-tRNAs. The ratio *k*_obs_/[Enzyme] gives the apparent deacylation efficiency of the enzyme.

| **Substrate (200 nM)** | **MmATD** | ***k*_obs_ (min^-1^)*** | ***k*_obs_/[Enzyme] (min^-1^ nM^-1^)** |
| --- | --- | --- | --- |
| **L-Tyr-tRNA^Tyr^** | 5 µM | No activity | - |
| **D-Tyr-tRNA^Tyr^** | 50 nM | 0.36 ± 0.03 | 0.0072 |
| **Gly-tRNA^Gly^** | 500 nM | 0.55 ± 0.04 | 0.0011 |
| **L-Ala-tRNA^Ala^** | 500 nM | 0.15 ± 0.03 | 0.0003 |
| **L-Thr-tRNA^Thr^** | 50 nM | 0.19 ± 0.05 | 0.0038 |
| **L-Ala-tRNA^Thr^** | **1 nM** | **0.27 ± 0.02** | **0.27** |
| **L-Ala-tRNA^Cys^** | 50 nM | 0.55 ± 0.05 | 0.011 |

* The deacylation graphs/curves for the calculation of *k*_obs_ have been taken from Figs. 4 and 5 as well as Supplementary Fig. 6.

**Supplementary Table 3 Comparative analysis of enrichment of tRNA^Thr^(G4•U69) genes and tRNA^Cys^(G4•U69) genes in representative organisms.** Relative abundance of tRNA^Thr^(G4•U69) genes and tRNA^Cys^(G4•U69) genes in representative organisms.

| **Organism** | **tRNA^Thr^ genes** | | | **tRNA^Cys^ genes** | | |
| --- | --- | --- | --- | --- | --- | --- |
|  | **Total** | **G4●U69** | **% abundance** | **Total** | **G4●U69** | **% abundance** |
| *Strongylocentrotus purpuratus** | 57 | 14 | **24.6** | 31 | 0 | **0** |
| *Latimeria chalumnae* | 22 | 5 | **22.7** | 21 | 2 | **10** |
| *Danio rerio* | 722 | 209 | **28.9** | 144 | 1 | **0.7** |
| *Takifugu rubripes* | 28 | 12 | **42.9** | 12 | 0 | **0** |
| *Xenopus tropicalis* | 245 | 74 | **30.2** | 83 | 2 | **2.4** |
| *Gallus gallus* | 10 | 4 | **40.0** | 10 | 0 | **0** |
| *Rattus norvegicus* | 16 | 4 | **25.0** | 40 | 0 | **0** |
| *Mus musculus* | 17 | 4 | **23.5** | 57 | 1 | **1.8** |
| *Pan troglodytes* | 18 | 4 | **22.2** | 27 | 0 | **0** |
| *Homo sapiens* | 20 | 4 | **20.0** | 29 | 1 | **3.4** |

* *Strongylocentrotus purpuratus* is the only organism known so far that does not belong to phylum Chordata (it belongs to phylum Echinodermata) but shows enrichment of tRNA^Thr^(G4•U69) genes.

**Supplementary Data 1 List of organisms whose genomes have been sequenced, highlighting the presence or absence of ATD.**

**Supplementary Data 2 Values of triplicate readings, their mean and standard deviation of biochemical assays.**
