# Supplementary material for: A chiral selectivity relaxed paralog of DTD for proofreading tRNA mischarging in Animalia
Source: Nat Commun. 2018 Feb 6;9:511. doi: 10.1038/s41467-017-02204-w (PMC5802732; doi:10.1038/s41467-017-02204-w)
Supplement: Supplementary file 2 — Description of Additional Supplementary Files [file 41467_2017_2204_MOESM2_ESM.pdf]

Descriptions of Additional Supplementary Files:

File Name: Supplementary Dataset 1
Description: List of organisms whose genomes have been sequenced, highlighting the presence or absence of ATD.

File Name: Supplementary Dataset 2
Description: Values of triplicate readings, their mean and standard deviation of biochemical assays.

File Name: Supplementary Movie 1
Description: Comparison between the active sites of DTD and ATD. Movie showing the change from Gly-*cis*Pro in DTD to Gly-*trans*Pro in ATD which creates “additional” space in the active site of ATD that can accommodate a larger group like methyl group as compared to just hydrogen in DTD. For ATD, the ligand was modeled in the active site after superposition of ATD dimer on PfDTD dimer (PDB id: 4NBI).

File Name: Supplementary Movie 2
Description: The flip from Gly-*cis*Pro in DTD to Gly-*trans*Pro in ATD. Movie depicting the remodeling of the local network of interactions due to *cis*-to-*trans* switch.
